# Biochemical Characterization of Recombinant UDPG-Dependent IAA Glucosyltransferase from Maize (*Zea mays*)

**DOI:** 10.3390/ijms22073355

**Published:** 2021-03-25

**Authors:** Anna Ciarkowska, Maciej Ostrowski, Anna Kozakiewicz

**Affiliations:** 1 Faculty of Biological and Veterinary Sciences, Nicolaus Copernicus University in Toruń, 87-100 Toruń, Poland; maciejost@umk.pl; 2 Faculty of Chemistry, Nicolaus Copernicus University in Toruń, 87-100 Toruń, Poland; akoza@umk.pl

**Keywords:** auxin conjugate, indole-3-acetic acid, UDPG-dependent IAA glucosyltransferase, enzyme modulators, inhibitor–enzyme interaction

## Abstract

Here, we report a biochemical characterization of recombinant maize indole-3-acetyl-β-d-glucose (IAGlc) synthase which glucosylates indole-3-acetic acid (IAA) and thus abolishes its auxinic activity affecting plant hormonal homeostasis. Substrate specificity analysis revealed that IAA is a preferred substrate of IAGlc synthase; however, the enzyme can also glucosylate indole-3-butyric acid and indole-3-propionic acid with the relative activity of 66% and 49.7%, respectively. *K_M_* values determined for IAA and UDP glucose are 0.8 and 0.7 mM, respectively. 2,4-Dichlorophenoxyacetic acid is a competitive inhibitor of the synthase and causes a 1.5-fold decrease in the enzyme affinity towards IAA, with the *K_i_* value determined as 117 μM, while IAA–Asp acts as an activator of the synthase. Two sugar-phosphate compounds, ATP and glucose-1-phosphate, have a unique effect on the enzyme by acting as activators at low concentrations and showing inhibitory effect at higher concentrations (above 0.6 and 4 mM for ATP and glucose-1-phosphate, respectively). Results of molecular docking revealed that both compounds can bind to the PSPG (plant secondary product glycosyltransferase) motif of IAGlc synthase; however, there are also different potential binding sites present in the enzyme. We postulate that IAGlc synthase may contain more than one binding site for ATP and glucose-1-phosphate as reflected in its activity modulation.

## 1. Introduction

Glycosyltransferases (GTs, EC (Enzyme Commission number) 2.4.x.y) are enzymes catalyzing transfer of a sugar moiety from an activated donor molecule to a wide range of acceptors with formation of glycoside linkages [1]. The majority of GTs are uridine diphosphate glycosyltransferases (UGTs) which utilize as a donor a nucleotide-activated sugar molecule (UDP sugar) [2]. Plant UGTs are cytosolic soluble proteins that share some structural similarities. They all contain a highly conserved PSPG (plant secondary product glycosyltransferase) motif located near the *C*-terminus of the protein. The PSPG motif is a 44 amino acid sequence which binds the UDP moiety of the UDP sugar substrate [3,4,5]. The *N*-terminus of the UGTs is a less conserved domain of the enzyme and binds the sugar acceptor molecule. The high sequence diversity of the *N*-terminal domain is responsible for the vast spectrum of substrates that are glycosylated by UGTs [4]. UGT-mediated glycosylation of plant compounds affects their biological activity, solubility and other properties [1]. Thus, phytohormone glycosylation is an important mechanism regulating plant hormonal homeostasis.

Auxins are phytohormones regulating proper growth and development of plants [6]. Local auxin concentration is modulated by different metabolic processes, such as de novo biosynthesis, degradation, hormone transportation and conjugation [7]. The latter results in the loss of biological auxinic activity of the phytohormone. In monocotyledonous plants, auxin is mostly conjugated to sugars or myo-inositol via ester bonds [8].

The ester conjugation pathway of indole-3-acetic acid (IAA), the main natural auxin, has been extensively studied in maize. The first step of ester–IAA conjugate synthesis is formation of 1-*O*-indole-3-acetyl-β-d-glucose (1-*O*-IAGlc) [9]. This reaction is catalyzed by IAA glucosyltransferase (1-*O*-IAGlc synthase) of the UGT family. As an energy-rich molecule, 1-*O*-IAGlc is subsequently utilized by various transferases as a donor of an indole-3-acetyl moiety for synthesis of other, more stable, IAA conjugates, such as indole-3-acetyl-myo-inositol [10,11].

Maize IAGlc synthase has been partially purified and characterized by Leźnicki and Bandurski [12,13]. Later, Kowalczyk and Bandurski [14] obtained a homogenous maize IAGlc synthase preparation which allowed identification of the *IAGLU* gene encoding this enzyme [15]. The authors confirmed identity of the *IAGLU* gene by its heterologous expression in *Escherichia coli*; however, characterization of the recombinant IAGlc synthase has not been performed. Few years later, glucosyltransferase UGT84B1 which conjugates glucose to IAA and other natural auxins was identified in *Arabidopsis thaliana* [16]. After that, a number of auxin glucosyltransferases has been identified in *A. thaliana* [17,18,19,20], as well as in other plant species, such as pea and rice [21,22]. Auxin ester conjugation has a great impact on a number of processes, such as seed development [23], seed germination [19], tiller formation [24], leaf positioning [25,26], hypocotyl elongation [20], gravitropic response [25], stress response [17,19] and more.

As the role of IAA UGTs in plant development and stress response seems significant, there is a need to deeply explore properties of these enzymes. Maize is a model monocotyledonous plant for studying ester–auxin conjugate synthesis; however, IAA UGTs are mostly explored in *A. thaliana*, a dicotyledonous plant which conjugates IAA mainly to amino acids. In this study, we produced recombinant maize IAGlc synthase and performed its biochemical characterization to complete studies started by Leźnicki and Bandurski [13] and provide deeper understanding of this enzyme vital for maintenance of auxin homeostasis.

## 2. Results and Discussion

### 2.1. Production of Recombinant Maize IAGlc Synthase in Escherichia coli and Purification of the Enzyme

**Figure 1:**
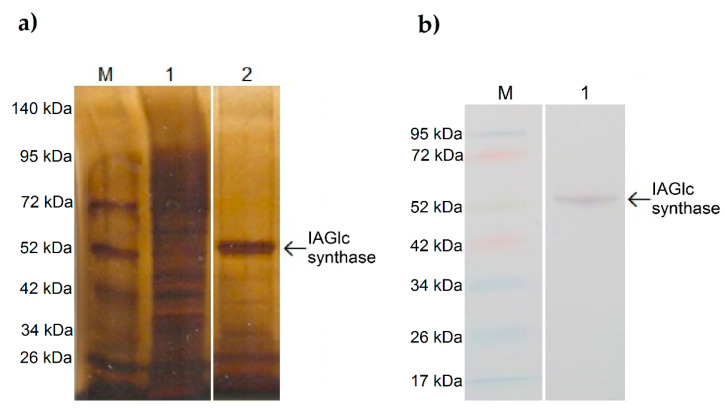
SDS-PAGE analysis of IAGlc synthase: M—molecular mass marker, 1—lysate, 2—purified IAGlc synthase fraction (**a**). Western blot analysis of IAGlc synthase: M—molecular mass marker, 1—purified IAGlc synthase fraction (**b**).

The *Zm-IAGLU* gene encoding maize IAGlc synthase was introduced into the *E. coli* BL21-CodonPlus(DE3)-RIL strain using the pET-15b expression vector provided by Antoni Leźnicki form the Department of Biochemistry at Nicolaus Copernicus University who performed cloning of the gene based on Szerszen et al. [15]. The activity of IAGlc synthase was detected in the soluble protein fraction of the lysate. Recombinant maize IAGlc synthase was *N*-terminally His-tagged; thus, we purified the protein from the lysate by Ni^2+^ affinity chromatography using an ÄKTA start chromatography system. IAGlc synthase was eluted as two peaks with 77 mM and 170.5 mM imidazole. The purity of obtained fractions was determined by SDS-PAGE analysis with silver staining. As shown in Figure 1a, an approximately 52 kDa-thick protein band which corresponds to His-tagged IAGlc synthase is present in the purified fraction. Western blot analysis with anti-polyHistidine antibodies (Figure 1b) confirmed that the ~52 kDa protein band contains a His-tag. The purified IAGlc synthase fraction shows trace presence of protein contaminants; however, the purity of the enzyme preparation was sufficient for kinetic studies.

### 2.2. Kinetic Properties of Maize Recombinant IAGlc Synthase

#### 2.2.1. Determination of pH Effect on IAGlc Synthase

**Figure 2:**
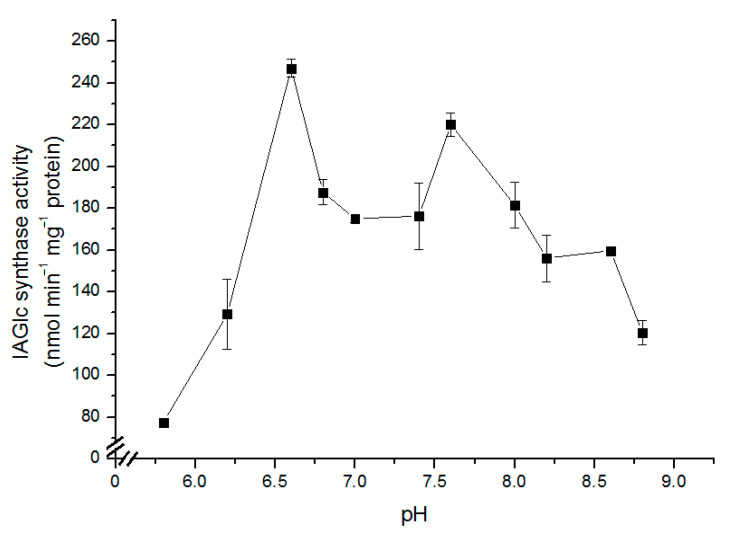
Effect of pH on the IAGlc synthase activity. Enzyme activity was determined using [^14^C]IAA after TLC (thin layer chromatography). For this assay, 25 mM HEPES buffers (pH 5.8; 6.2; 6.6; 6.8; 7.0; 7.4; 7.6; 8.0; 8.2; 8.6 and 8.8) were used. Catalytic activity was expressed as the means ± SD for three repetitions.

The effect of pH on the IAGlc synthase activity was determined in the pH range from 5.8 to 8.8 (pH 5.8; 6.2; 6.6; 6.8; 7.0; 7.4; 7.6; 8.0; 8.2; 8.6; 8.8) as shown in Figure 2. The enzyme shows high activity (above 100 nmol min^−1^ mg^−1^ protein) in a broad pH range (pH 6.2–8.8). Catalytic activity of IAGlc synthase visibly decreases at pH lower than 6.0. The highest activity was observed at pH 6.6 and 7.6. The optimal pH for native IAGlc synthase activity was determined to be 7.1 by Michalczuk and Bandurski [27] and 7.3–7.6 by Leźnicki and Bandurski [12], which is similar to the second activity peak of the recombinant enzyme. Interestingly, native IAGlc synthase exhibited no activity at pH lower than 6.7 [12], while the recombinant enzyme displays the highest activity at pH 6.6 and starts losing catalytic activity at pH below 6.0. This difference could be a result of different folding conditions occurring in bacterial and plant cells [28]. UGT74D1, an IAA glycosyltransferase from *A. thaliana*, is also active in the broad pH spectrum (pH 6.0–9.0) when the HEPES (4-(2-hydroxyethyl)-1-piperazineethanesulfonic acid) buffer is used, with the highest catalytic activity at pH 6.0 and 7.0 [29]. Similar to maize IAGlc synthase, UGT74D1 exhibits rapid decrease in activity at pH lower than 6.0. *A. thaliana* UGT75D1 was most active at pH 7.0 with most of the tested buffers; however, it also showed high activity at pH 6.0 and 9.0 [19]. At pH 5.0 and 9.0, UGT75D1 exhibited no catalytic activity with most of the tested buffers. Rice OsIAGT1 is also active at pH ranging from 6.0 to 9.0, with the highest activity at pH 8.0 and with the Tris buffer [22]. HEPES was not used for determination of the optimal pH of this enzyme. Catalytic activity of UGTs is often dependent on the buffer used for analysis. As for native maize IAGlc synthase, the enzyme exhibited similar activity with both HEPES and Tris buffers; however, it was strongly inhibited by the 100 mM phosphate buffer [12]. UGTs are active in the broad pH spectrum and usually display the highest catalytic activity at pH close to 7.0, which is consistent with their cytoplasmic localization [5,26]. It should be noted that another IAA-conjugating enzyme, IAA–aspartate synthetase GH3 from pea seeds is also localized in the cytoplasm [30].

#### 2.2.2. Determination of IAGlc Synthase Substrate Specificity

IAA is the main natural auxin; however, other auxinic hormones are also present in plants and can form ester conjugates with glucose. As determined by Leźnicki and Bandurski [13], native maize IAGlc synthase catalyzes conjugation of IAA to glucose and does not utilize oxidized forms of IAA, oxindole-3-acetic acid (OxIAA) and 7-OH-OxIAA, as sugar acceptors. Kowalczyk et al. [31] analyzed various non-indolic auxins and aromatic compounds as potential glucose acceptors of native maize IAGlc synthase. Based on these results, IAGlc synthase effectively conjugated 1-naphtaleneacetic acid (1-NAA) and phenylacetic acid (PAA) to their glucose conjugates (more than 70%, and 30% of the relative activity in comparison to IAA, respectively). 2,4-Dichlorophenoxyacetic acid (2,4-D) was a poor acceptor of a glucosyl moiety.

We have tested some natural auxins as well as synthetic auxinic herbicides as potential sugar acceptors of maize glucosyltransferase (Table 1). Recombinant IAGlc synthase exhibits the highest activity towards IAA; however, it can also utilize indole-3-butyric acid (IBA) and indole-3-propionic acid (IPA) as glucose acceptors, though with lower activity (relative activity is 66% and 49.7%, respectively). Only trace glucosyltransferase activity was detected towards indole-3-pyruvic acid (IPyA) (relative activity of 2.76%). PAA and tryptophan, an amino acid which contains the indole ring characteristic of IAA, were not substrates for IAGlc synthase. Moreover, IAGlc synthase showed no catalytic activity towards synthetic auxins, 2,4-D, Picloram and Dicamba. IAA is also a preferred substrate of pea IAA glucosyltransferase; however, the enzyme can also glucosylate other auxins, IPA and 1-NAA, with the relative activity of 22% and 24%, respectively [21]. *A. thaliana* UGT84B1 has similar affinity towards IAA, IBA and IPA; however, IAA is the preferred substrate of the enzyme [16,32]. Another *A. thaliana* UGT, UGT74E2, strongly favors IBA as a substrate; however, it can also glucosylate, with less efficiency, IAA, 2,4-D, NAA and some other phytohormones [17]. Rice OsIAGT1 exhibits high catalytic activity towards IBA, IPA and IAA and lower activity towards NAA and 2,4-D [22]. The highest preference towards IBA is also characteristic of *A. thaliana* UGT74D1 [29]. This enzyme can also quite efficiently glucosylate other auxins, such as IAA, IPA, NAA and, less effectively, 2,4-D. Additional studies revealed that UGT74D1 can also utilize OxIAA as a glucose acceptor and has higher affinity to this substrate compared to IAA [18].

Even though most auxinic UGTs have a relatively broad substrate specificity and, like maize IAGlc synthase, can utilize more than one glucose acceptor, there are enzymes which glucosylate only one type of auxin. *A. thaliana* UGT76F1 catalyzes conjugation of IPyA, but not of IAA and IBA, despite the latter two being the dominant forms of auxin present in the plant [20]. Moreover, *A. thaliana* UGT75D1 shows only trace activity towards IAA, IPA and NAA, glucosylating almost exclusively IBA [19].

#### 2.2.3. Determination of Kinetic Parameters of IAGlc Synthase

**Figure 3:**
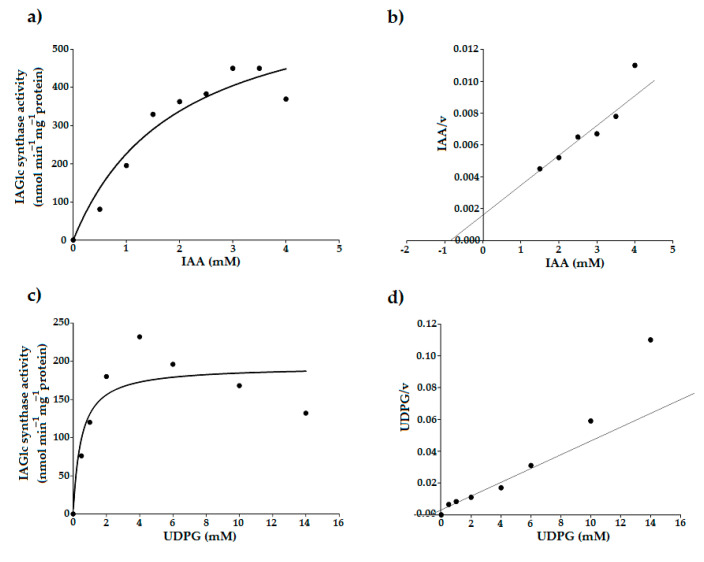
Determination of kinetic parameters of IAGlc synthase. Effect of IAA concentration on the IAGlc synthase activity: Michaelis–Menten plot (**a**) and Hanes–Woolf plot (**b**). Effect of UDPG concentration on the IAGlc synthase activity: Michaelis–Menten plot (**c**) and Hanes–Woolf plot (**d**). The *K_M_* and *V_max_* values were calculated using Hanes–Woolf plots (IAA/v = f_(IAA)_ and UDPG/v = f_(UDPG)_, respectively).

Maize IAGlc synthase displays the Michaelis–Menten hyperbolic kinetic characteristic for isosteric enzymes (Figure 3a,c). Leźnicki and Bandurski [12] failed to determine *K_M_* for UDPG of native maize IAGlc synthase as the enzyme showed nonlinear kinetics. The authors calculated approximate affinity of the synthase to be 14 mM and postulated that nonlinear kinetics is an effect of the absence of the potential cofactor. Later, it was revealed that IAGlc synthase requires presence of divalent cations which were not used for *K_M_* determination [13]. Thus, the native IAGlc synthase *K_M_* for IAA, which was determined to be 1 mM in the absence of divalent cations [12], is also questionable. However, it is very similar to *K_M_* for IAA of the recombinant enzyme, which is 0.8 ± 0.1 mM (Table 2) as determined using a Hanes–Woolf plot (Figure 3b). Similar values of *K_M_* for IAA suggest that divalent cations are not required for binding of the sugar acceptor but only for binding of the sugar donor. *K_M_* for UDPG determined using a Hanes–Woolf plot (Figure 3d) for the recombinant enzyme in the presence of Mg^2+^ was 0.7 ± 0.09 mM (Table 2), which indicates that divalent cations increase affinity of the IAGlc synthase for UDPG. Other UGTs which prefer IAA as a substrate have similar *K_M_* values to IAGlc synthase, that is 0.52 mM for IAA glucosyltransferase from pea [21] and 0.24 mM for *A. thaliana* UGT84B1 [16]. However, pea IAA glucosyltransferase has *K_M_* = 2.67 mM for UDPG [21], which indicates lower affinity for the glucose donor compared to maize IAGlc synthase.

#### 2.2.4. Effect of Divalent Cations on IAGlc Synthase

**Figure 4:**
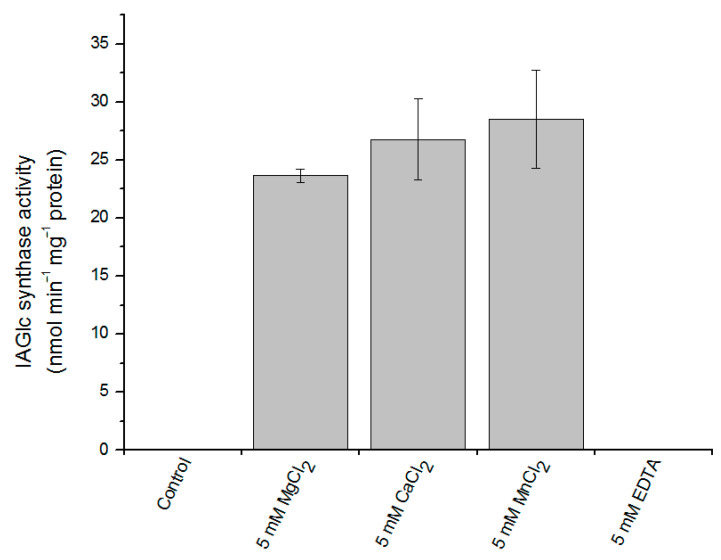
Effect of divalent cations on IAGlc synthase. Enzyme activity was determined using [^14^C]IAA after TLC in the presence of 5 mM MgCl_2_, 5 mM CaCl_2_, 5 mM MnCl_2_ or 5 mM EDTA; the control mixture did not contain any divalent cations. Catalytic activity was expressed as the means ± SD for three repetitions.

UGT activity can be modulated by a number of different compounds. Maize IAGlc synthase requires presence of divalent cations to display its catalytic activity [13]. The recombinant enzyme shows no activity if divalent cations are not present in the reaction mixture or when a chelating agent, EDTA, is added (Figure 4). The highest activity of the enzyme was observed in the presence of Mn^2+^ ions and a little less active when the reaction mixture contained Ca^2+^ and Mg^2+^; this is in agreement with the native enzyme properties [13].

#### 2.2.5. Effect of Modulators on IAGlc Synthase

**Figure 5:**
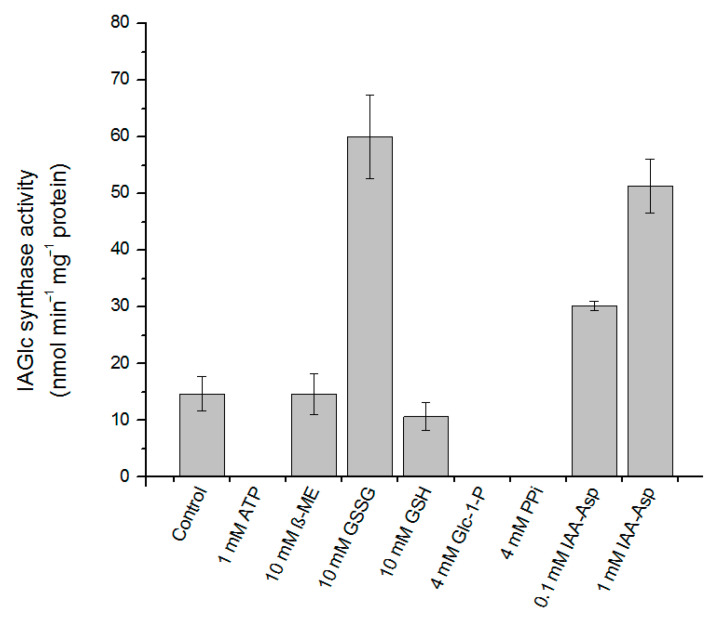
Effect of modulators on IAGlc synthase. Enzyme activity was determined using [^14^C]IAA after TLC; the control mixture did not contain any modulators. Catalytic activity was expressed as the means ± SD for three repetitions. ATP—adenosine triphosphate; β-ME—β-mercaptoethanol; GSSG—oxidized glutathione; GSH—reduced glutathione; Glc-1-P—glucose-1-phosphate; PP_i_—pyrophosphate; IAA–Asp—indole-3-acetyl-aspartate.

Some compounds act as activators of recombinant maize IAGlc synthase (Figure 5). An amide IAA conjugate, IAA–Asp, affected activity of the enzyme in a concentration-dependent manner. IAA–Asp in the amount of 0.1 mM increased catalytic activity of IAGlc synthase approximately twofold, while in the presence of 1 mM conjugate, the activity of the enzyme increased 3.5-fold. The activating effect of IAA–Asp may have deeper implications. IAA–Asp is synthesized by amido synthetases from the GH3 family (the expression thereof is auxin-inducible) [8]. Thus, an elevated level of IAA–Asp signalizes a high level of endogenous IAA and may indicate a need in a more effective reduction of the free auxin pool, for example, by its ester conjugation. IAGlc synthase activation by IAA–Asp may act as the means of better control of auxin homeostasis. For verification of this hypothesis, we studied the effect of 0.1 mM IAA–Asp on the *V_max_* value and affinity of IAGlc synthase for IAA (defined by the *K_M_* value). Surprisingly, kinetic studies revealed that IAA–Asp enhanced *V_max_* about 1.6-fold, but also increased the *K_M_* value (approximately 2.4-fold). This finding may suggest that IAA–Asp competes with IAA for the catalytic site of IAGlc synthase and reduces affinity of the enzyme for IAA. Considering that IAGlc synthase catalyzes a two-substrate reaction, IAA–Asp might potentiate catalytic efficiency by an unknown mechanism.

The activating effect on recombinant IAGlc synthase is also displayed by oxidized glutathione (GSSG) at 10 mM concentration, which activated the enzyme fourfold (Figure 5). Interestingly, reduced glutathione (GSH) at the same concentration is an inhibitor of the enzyme (relative activity of 72%). In the study on the native enzyme, GSH acted as an activator of the synthase [13].

Maize IAGlc synthase contains eight cysteine residues in the amino acid sequence. Analysis performed with the DiANNA (DiAminoacid Neural Network Application) 1.1 web server revealed that Cys103 is highly likely to form a disulfide bond with Cys360. The recombinant enzyme obtained in bacterial cells can exhibit some structural differences from the native protein synthesized in plant cells caused by disturbance in disulfide bond formation [33]. The further evidence that the expression host may affect protein properties is that β-mercaptoethanol (β-ME), another reducing agent, has no effect on recombinant IAGlc synthase (Figure 5) but is an activator of the native enzyme [13].

**Figure 6:**
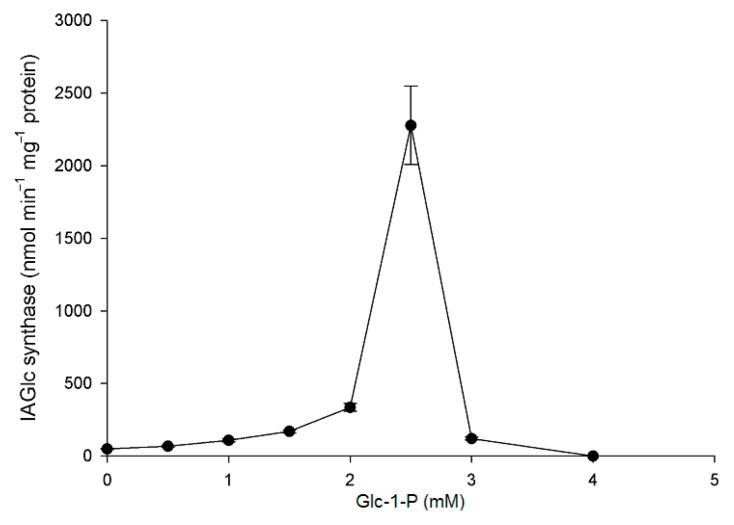
Effect of glucose-1-phosphate (Glc-1-P) on IAGlc synthase. The following concentrations of Glc-1-P were used for this assay: 0; 0.5; 1.0; 1.5; 2.0; 2.5; 3.0 and 4.0 mM. Enzyme activity was determined using [^14^C]IAA after TLC. Catalytic activity was expressed as the means ± SD for three repetitions.

**Figure 7:**
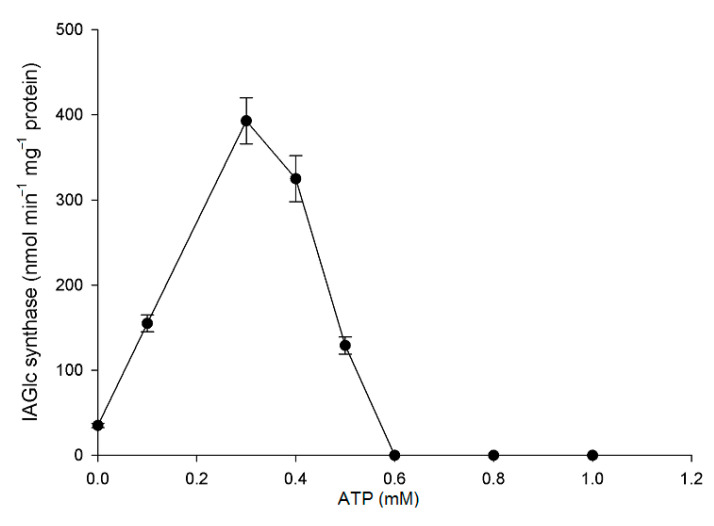
Effect of ATP on IAGlc synthase. The following concentrations of ATP were used for this assay: 0; 0.1; 0.3; 0.4; 0.5; 0.6; 0.8 and 1.0 mM. Enzyme activity was determined using [^14^C]IAA after TLC. Catalytic activity was expressed as the means ± SD for three repetitions.

Of the tested compounds, three have a completely inactivated recombinant IAGlc synthase: 1 mM ATP, 4 mM pyrophosphate (PP_i_) and 4 mM glucose-1-phosphate (Glc-1-P) (Figure 5). This is in agreement with the inhibitory effect of phosphate compounds, such as PP_i_, phosphate esters and phospholipids, reported before and with inhibition by the phosphate buffer [13]. On the contrary, *A. thaliana* UGT84B1 was not inhibited by phospholipids [16]. Interestingly, when effect of different concentrations of glucose-1-phosphate on recombinant maize IAGlc synthase was tested, at lower concentrations, it had an activating effect on the enzyme (Figure 6). IAGlc synthase activity increased rapidly in the presence of 2.5 mM glucose-1-phosphate, but at 3 mM, it was on a similar level to the control. As mentioned above, a higher 4 mM concentration of glucose-1-phosphate completely abolished IAGlc activity. It is well-known that glucose-1-phosphate is not a donor of a glucosyl moiety for glucosyltransferase activity, but is involved in UDPG biosynthesis according to the reaction catalyzed by UDPG pyrophosphorylase: glucose-1-phosphate + UTP ↔ UDPG + PP_i_. In this study, we analyzed enzyme activity of UDPG pyrophosphorylase in the purified fraction of IAGlc synthase. No UDPG-synthesizing activity was observed (data not shown). A similar effect was also observed for ATP, though its activating effect was more prominent in a wider concentration range (Figure 7). ATP activated IAGlc synthase at 0.1–0.5 mM concentrations with the activity peak observed in the presence of 0.3 mM ATP. When the concentration used was 0.6 mM or higher, ATP completely abolished the IAGlc synthase activity. UGTs utilize UDP glucose as a sugar donor, therefore we propose that at higher concentrations, ATP competes with the nucleotide moiety of UDPG for binding to the PSPG motif of the enzyme. IAGlc synthase may possess two binding sites for ATP; one of them is specific, to which ATP binds and activates the enzyme, and the PSPG motif, where ATP binds as a competitive inhibitor. On the other hand, it cannot be excluded that IAGlc synthase has one binding site of UDPG/ATP and higher concentrations of ATP block this site and reduce the catalytic activity. It is possible that the mechanism of IAGlc synthase activity modulation by glucose-1-phosphate is similar to that of ATP, as both glucose and phosphate are present in UDPG. Thus, high glucose-1-phosphate concentration may block binding of UDPG to the enzyme.

#### 2.2.6. Effect of Phytohormones on IAGlc Synthase

**Figure 8:**
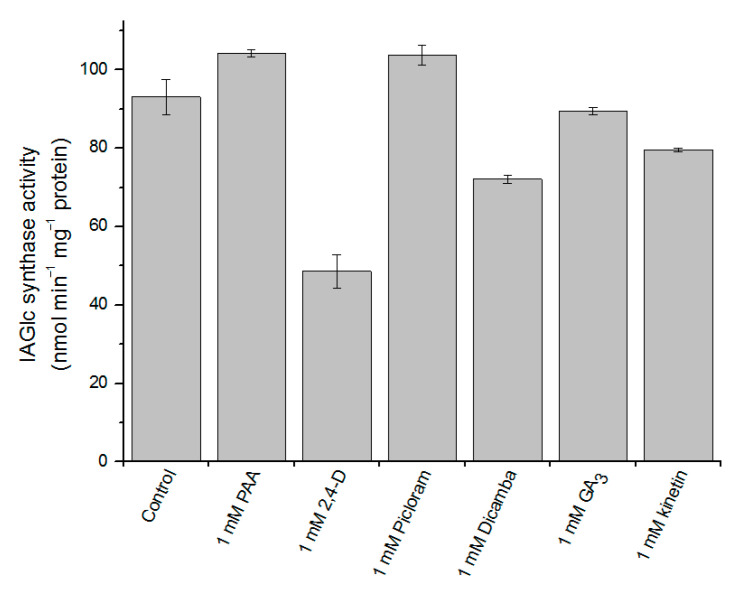
Effect of phytohormones on IAGlc synthase. Phytohormones that are not utilized by IAGlc synthase as substrates were used for this assay. Enzyme activity was determined using [^14^C]IAA after TLC; the control mixture did not contain any additional phytohormones. Catalytic activity was expressed as the means ± SD for three repetitions. PAA–phenylacetic acid; 2,4-D—2,4-dichlorophenoxyacetic acid; GA_3_—gibberellic acid.

The IAA glucosyltransferase activity may also be modulated by other phytohormones. Pea IAA glucosyltransferase is inhibited by IBA, which is not a substrate of this enzyme but only acts as a competitive inhibitor [21]. Native maize IAGlc synthase is also inhibited by some phytohormones: zeatin, 2,4-D, gibberellic acid (GA), abscisic acid and kinetin [13]. However, cytokinin had no inhibitory effect on *A. thaliana* UGT84B1 [16]. Maize IAGlc synthase was also inhibited by IAA conjugates: indole-3-acetyl-myo-inositol and IAA-β-1,4-glucan. As IAGlc synthase is the first enzyme of the IAA ester conjugation pathway, it is inhibited by the final products of auxin conjugation. As the phytohormonal effect on IAA glucosyltransferases is evident, we tested some plant hormones as potential recombinant IAGlc synthase inhibitors (Figure 8). We confirmed that GA_3_ and kinetin have a slight inhibitory effect on the enzyme, while 2,4-D strongly decreases the IAGlc synthase activity.

**Figure 9:**
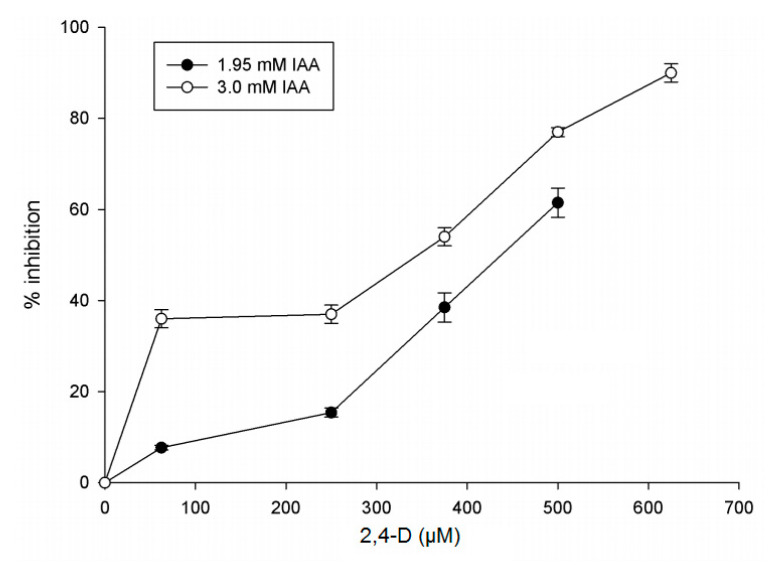
Effect of 2,4-D (2,4-dichlorophenoxyacetic acid) on IAGlc synthase. The assay was performed in the presence of 1.95 or 3.0 mM IAA. Inhibition (%) was expressed as the means ± SD for three repetitions. The following concentrations of 2,4-D were used for this assay: 0; 75; 250; 375; 500 and 675 μM. Enzyme activity was determined using [^14^C]IAA after TLC.

We also tested effect of PAA and auxinic herbicides Dicamba and Picloram on the enzyme. While PAA and Picloram slightly activated IAGlc synthase, Dicamba turned out to be another inhibitor of the enzyme. As 2,4-D exhibited the strongest inhibitory effect on IAGlc synthase, we investigated its effect in a concentration-dependent manner in the presence of 1.95 mM and 3 mM IAA (Figure 9). 2,4-D had a negative effect on the IAGlc synthase activity at all tested concentrations, with the rapid increase in inhibition above the 250 µM concentration. The 2,4-D IC_50_ (50% inhibition) values for 1.95 mM and 3 mM IAA were 439 and 350 µM, respectively (Table 3). We determined that 2,4-D displays a competitive type of inhibition as it is structurally similar to IAA and causes an approximately 1.5-fold decrease in affinity of the enzyme towards IAA (Table 3).

The inhibition type of the enzymatic reaction was determined through the graphical method using a Dixon plot. The inhibition constant (*K_i_*) value was calculated using a Dixon plot. The IC_50_ parameter defines an inhibitor concentration that causes 50% inhibition of the enzyme activity and was calculated through the graphical method. The resulting data were fit into the following equation: *K_M_^app^* = *K_M_* (1 + *[I]/K_i_*). All the values were expressed as the means ± SD (*n* = 3).

### 2.3. Sequence Analysis, 3D Structure Prediction and Molecular Docking Studies of Maize IAGlc Synthase

**Figure 10:**
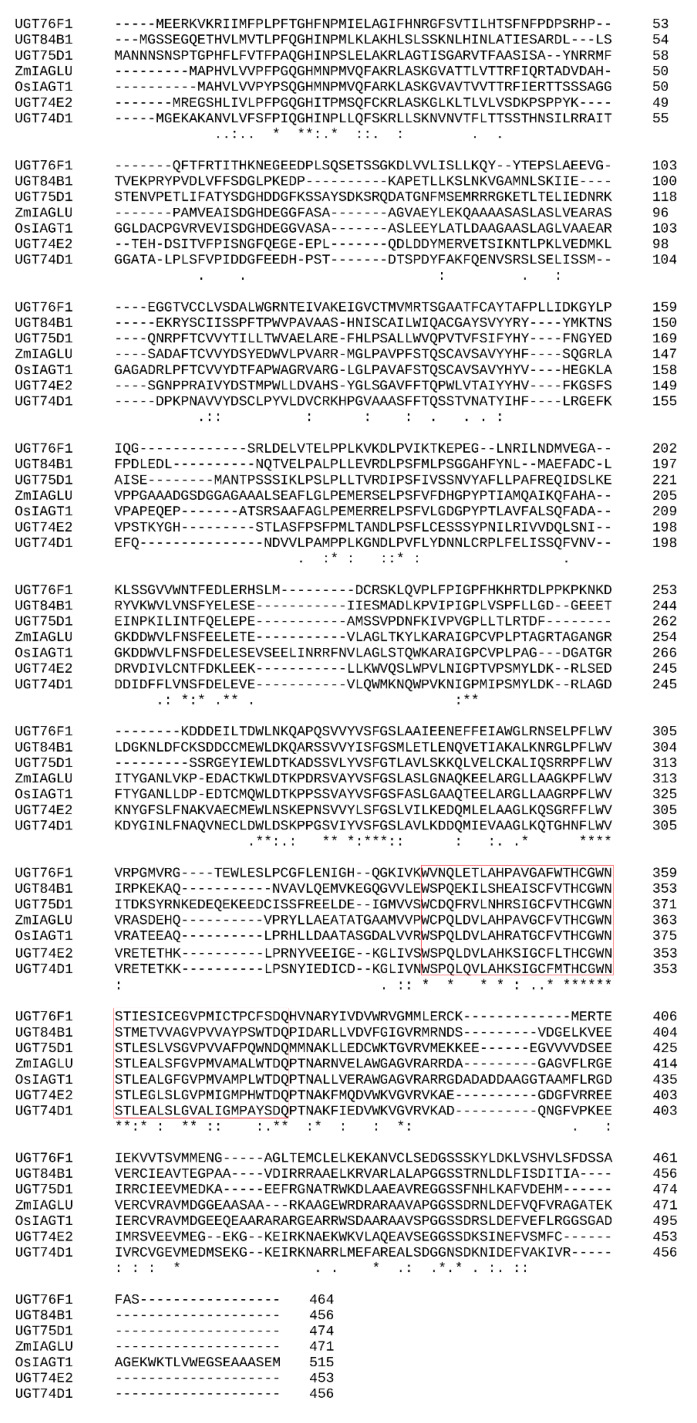
Sequence alignment of IAA glucosyltransferases: maize IAGlc synthase (ZmIAGLU, NCBI Reference Sequence Database, accession number NP_001105326.1), rice OsIAGT1 (GenBank, accession number BAS85861.1) and *A. thaliana* glucosyltransferases UGT74D1 (UniProtKB/Swiss-Prot, accession number Q9SKC5.1), UGT74E2 (GenBank, accession number ABJ17124.1), UGT75D1 (UniProtKB/Swiss-Prot, accession number O23406.2), UGT76F1 (UniProtKB/Swiss-Prot, accession number Q9M051.1) and UGT84B1 (GenBank, accession number AAO63432.1). The red box indicates the PSPG motif. The sequence alignment was generated using Clustal Omega. (‘*’—positions with a single, fully conserved residue; ‘:’—conservation between groups of strongly similar properties, scoring > 0.5 in the Gonnet PAM (Percent Accepted Mutation) 250 matrix; ‘.’—conservation between groups of weakly similar properties, scoring ≤ 0.5 in the Gonnet PAM 250 matrix.

Using Clustal Omega, we aligned IAGlc synthase with other IAA glucosyltransferases (Figure 10). Clustal Omega analysis revealed that maize IAGlc synthase (ZmIAGLU) shows a 69.35% identity with OsIAGT1 (Table 4). Both UGTs are from monocotyledonous plants and utilize IAA and IBA as glucose acceptors [22], hence high sequence identity. Maize IAGlc synthase shows a definitely lower identity to dicotyledonous *A. thaliana* UGTs. The lowest sequence identity (27.55%) is shared with UGT76F1, which catalyzes glucosylation of IPyA, an IAA precursor, but does not utilize IAA or IBA as substrates [20]. Maize IAGlc synthase catalyzes glucosylation of IAA, IBA and IPA with a higher preference towards the first auxin (Table 1). Other *A. thaliana* UGTs, UGT74D1, UGT74E2, UGT75D1 and UGT84B1, similar to maize IAGlc synthase, utilize both IAA and IBA as substrates [17,19,29,32], hence higher sequence identity, which is 39.55%, 40.41%, 35.27% and 34.10%, respectively (Table 4).

The PSPG (plant secondary product glycosyltransferase) motif is the most conserved part of the GT sequence [4,34]. The identity of ZmIAGLU and OsIAGT1 PSPG motifs is 88.64% (Table 4). Even with the least similar UGT76F1, maize IAGlc synthase has a 59.09% identity when only the PSPG motif is concerned. The PSPG motif consists of 44 amino acid residues that participate in binding of the sugar nucleotide substrate. The most conserved residues of the PSPG motif are Trp (W1 and 22), Gln (Q4 and 44), Leu (L8), His (H10 and 19), Cys (C20), Gly (G21), Asn (N23), Ser (S24), Glu (E27), Pro (P39) and Glu/Asp (E/D43) (numbers in the brackets indicate the amino acid position in the PSPG motif). Those residues are conserved in all analyzed IAA UGTs, with the exception of proline P39, which in maize IAGlc synthase is substituted with alanine. The last residue of the PSPG motif is crucial for sugar donor specificity of GTs. GTs which utilize glucose nucleotides have glutamine in this position (Q44), while activity required for recognition of the galactose moiety is determined by histidine (H44) [35]. Substitution of Q44 by the His residue caused *Vitis vinifera* UGT78A12 to switch activity form glucosyltransferase to galactosyltransferase. All IAA UGTs catalyze formation of the IAA–glucose conjugate; thus, they have glutamine as the last residue of the PSPG motif. Moreover, activity analysis of native maize IAGlc synthase revealed that the enzyme is strictly specific for UDP glucose and shows no catalytic activity towards UDP galactose or UDP xylose [13].

**Figure 11:**
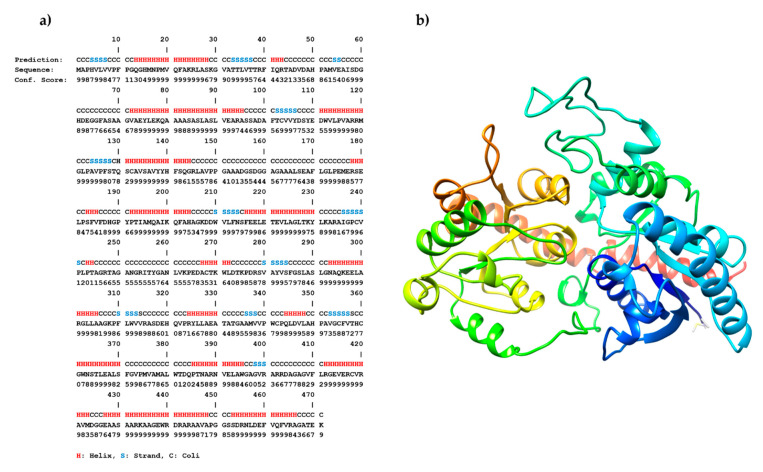
Three-dimensional structure of IAGlc synthase was modeled with the I-TASSER server (https://zhanglab.ccmb.med.umich.edu/I-TASSER/, accessed on 20 February 2021) [36,37,38,39]. The sequence-based prediction of the secondary structure using I-TASSER/PSSpred. The helix is marked in red, the strand is blue, and the coil is black (**a**). Predicted 3D model of IAGlc synthase in ribbon-style representation (**b**).

Homology modeling of IAGlc synthase was carried out on the iterative threading assembly refinement (I-TASSER) server (Figure 11). For the obtained model, the root-mean-square deviation (RMSD) and the TM-score (index for assessing the topological similarity of model and template) were 5.5 ± 3.5 Å and 0.82 ± 0.09, respectively. The C-score, which gives the confidence for the quality of the predicted models (C-score is typically in the range of (–5, 2)), was estimated to be 0.77. This 3D model of IAGlc synthase was used in molecular docking.

**Figure 12:**
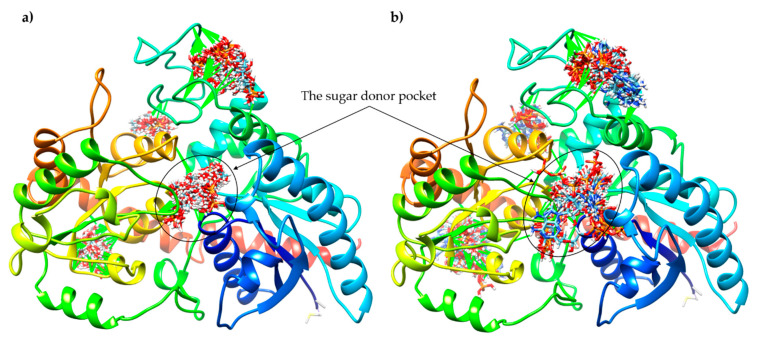
Results of docking of Glc-1-P (**a**) and ATP (**b**) to IAGlc synthase performed with the SwissDock server.

In silico molecular docking was performed with two different computational programs, SwissDock [40] and AutoDock Vina [41], to study the interaction of ATP and Glc-1-P with IAGlc synthase. We performed simple docking experiments in order to predict a possible binding site of ATP and Glc-1-P to IAGlc synthase, with the use of the SwissDock Server. Results indicated four different binding sites of these modulators to the protein (Figure 12). This may explain the different modulation (inhibition or activation) of the IAGlc synthase activity by these two compounds. One of the highest-ranked regions of ATP as well as Glc-1-P binding to IAGlc synthase is located in the sugar donor pocket. This pocket is the binding site of UDPG, therefore, we postulate that the modulator binding at this site decreases the activity of the synthase.

The IAGlc synthase possesses a highly conserved PSPG motif (plant secondary product glycosyltransferase), which is located on one side of the sugar donor pocket. Amino acids of the PSPG motif participate in binding interactions with the sugar donor (UDPG). There are 10 amino acids in this motif that play an important role in the binding interaction with the sugar donor substrate (W341, C342, P343, H359, W362, N363, S364, E367, D383 and Q384) [4]. Data on the structure of the substrate binding site of IAGlc synthase allowed for docking studies of UDPG and its modulators (ATP, Glc-1-P). The semiflexible molecular docking using AutoDock was performed. The protein molecule was kept rigid and the grid box was fixed around the sugar donor pocket of the enzyme which possessed the PSPG motif, whereas UDPG, ATP and Glc-1-P were kept as flexible molecules.

**Figure 13:**
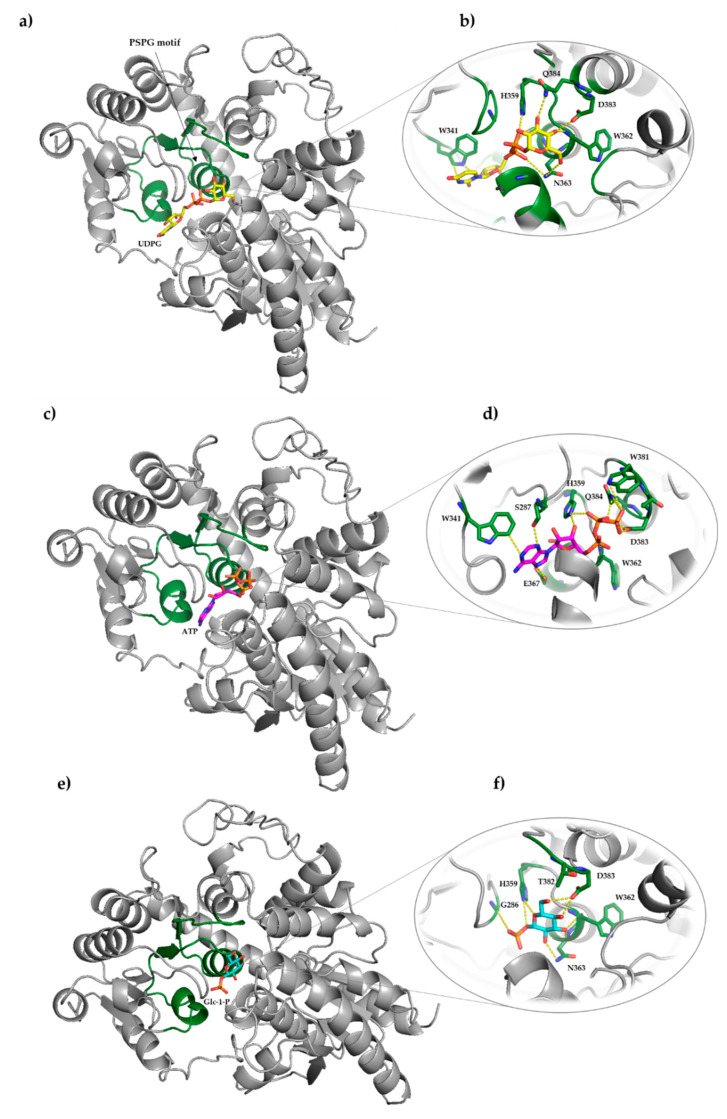
Docking analysis visualization of UDPG (**a**), ATP (**c**) and Glc-1-P (**e**) to IAGlc synthase performed with AutoDock. Hydrogen bonding interactions of UDPG (**b**), ATP (**d**) and Glc-1-P (**f**) with amino acid residues of the enzyme. Possible hydrogen bonds are marked with yellow dashed lines. The PSPG motif is presented in the ribbon-style representation in green.

Molecular docking results revealed that the enzyme interacts with UDPG, ATP and Glc-1-P mainly through residues of the PSPG motif (Figure 13). The uracil ring of UDP forms interactions with the indole ring of W341 (Figure 13b). Similar interactions are also observed in the complex structure of IAGlc synthase with ATP. The purine ring of ATP is involved in interactions with W341. Moreover, the hydrogen bonds between the nitrogen atoms of adenine and the oxygen atom of S287 and E367 were detected (Figure 13d). The glucose moiety of UDPG forms hydrogen bonds with the nitrogen atom of N363 and Q384 and the oxygen atom of D383. Furthermore, contacts between nitrogen of the W362 main chain and glucose were observed. The α-phosphate of UDPG forms hydrogen bonds through its oxygen atom and the N atom of N363, while β-phosphate interacts through its oxygen atom with the N atom of H359. In the case of ATP binding, the several interactions between phosphates and the enzyme were also found. The hydrogen bond between the oxygen atom of α-phosphate and nitrogen of the W362 main chain was observed. Moreover, β-phosphate makes contacts with the enzyme through hydrogen bonds between its nitrogen atoms of H359 and Q384. What is more, the nitrogen atom of H359 participates in the H-bond with the oxygen atom of the ribose ring. Furthermore, the oxygen atoms of the γ-phosphate form interactions with the nitrogen atom of W381 and the oxygen atom of D383. For Glc-1-P, the interactions between the glucose ring and four residues (W362, N363, D383 and H359) were detected (Figure 13f). At the same time, α-phosphate forms interactions with the N atom of H358 and the main chain’s nitrogen atom.

## 3. Materials and Methods

### 3.1. Expression of Zm-IAGLU in E. coli and Purification of Recombinant IAGlc Synthase

We received the pET-15b-*Zm-IAGLU* vector used for the IAGlc synthase expression from Antoni Leźnicki from the Department of Biochemistry of Nicolaus Copernicus University who performed cloning of the gene into the expression vector based on Szerszen et al. [15]. The pET-15b-*Zm-IAGLU* bacterial expression construct was transformed into *E. coli* BL21-CodonPlus(DE3)-RIL. Bacteria cells were grown at 37 °C in an LB medium containing 50 μg/mL ampicillin and 0.5% (*w/v*) glucose until OD_600_ = 0.4. Protein synthesis was induced by addition of isopropyl-1-thio-β-D-galactopyranoside (IPTG) to a final concentration of 0.2 mM and the culture was continued for 20 h at 18 °C. The cells were pelleted by centrifugation (3500× *g*, 20 min, 4 °C, Sigma Sartorius Centrifuge, Goettingen, Germany) and resuspended in 25 mM HEPES buffer, pH 7.4, containing 0.5 M NaCl, 30 mM imidazole and 10% (v/v) glycerol (buffer A). The cells were lysed by sonication (4 × 20 s, Ultrasonic Disintegrator UD-20, Techpan, Warszawa, Poland). The lysate was centrifuged at 10,000× g for 10 min at 4 °C (Sigma Sartorius Centrifuge). Recombinant IAGlc synthase carrying *N*-terminal His-tag was purified from the supernatant using an ÄKTA^TM^ start system (GE Healthcare Bio-Sciences, Uppsala, Sweden). The supernatant was loaded into a His-Trap^TM^ HP column pre-packed with the Ni Sepharose™ High Performance medium (GE Healthcare Bio-Sciences) previously equilibrated with buffer A. The unbound proteins were removed by washing the column with buffer A. IAGlc synthase was eluted with 77 mM and 170.5 mM imidazole in 25 mM HEPES buffer, pH 7.4, containing 0.5 M NaCl and 10% (v/v) glycerol. The preparation was concentrated using an Amicon-ultra 15 centrifugal filter unit (Millipore, Burlington, Massachusetts, USA) according to the manufacturer’s instructions. Purity of the enzyme preparation was examined by SDS-PAGE [42] with silver nitrate staining [43]. The fraction of the highest purity was used for further analysis.

### 3.2. Enzyme Activity Assay

#### 3.2.1. Determination of IAGlc Synthase Activity

IAGlc synthase activity was determined in a total volume of 8 μL containing 25 mM HEPES buffer, pH 7.4, 7.5 mM UDPG, 4 mM IAA, 0.016 μCi [2′-^14^C]IAA (55 mCi mmol^−1^, Hartmann Analytic GmBH, Braunschweig, Germany) and 2.5 mM MgCl_2_ with 3 μL of the enzyme preparation. The reaction was stopped after 15 min incubation at 30 °C by drying 4 μL of aliquots on a Silica Gel F_260_ TLC plate (Merck, Darmstadt, Germany). TLC was performed using ethyl acetate: n-butanone: ethanol: water (5/3/1/1, *v/v/v/v*) as a solvent. The indole ring compounds were visualized by staining the plate with the Van Urk–Salkowski reagent [44]. Bands identified as IAGlc were excised and placed in a vial with 2 mL EcoLite (+) scintillation fluid (ICN). The radioactivity level was measured using a Wallac 1409 liquid scintillation counter (Turku, Finland).

#### 3.2.2. Determination of UDPG Pyrophosphorylase Activity

UDPG pyrophosphorylase (1) activity was analyzed using the spectrophotometric method using a coupled enzyme assay containing phosphoglucomutase (2) and glucose-6-phosphate dehydrogenase (3) according to the following equations:

UDPG + PP_i_ ↔ UDP + glucose-1-phosphate (1)

glucose-1-phosphate ↔ glucose-6-phosphate(2)

glucose-6-phosphate + NADP^+^ → 6-phosphogluconolactone + NADPH/H^+^ (3)

The reaction mixture in the total volume of 1.0 mL contained 0.2 M HEPES–NaOH, pH 7.4, 6 mM MgCl_2_, 2 mM sodium pyrophosphate, 10 mM UDPG, 0.4 mM NADP ^+^, 3 U glucose-6-phosphate dehydrogenase (Sigma-Aldrich, Saint Louis, MO, USA), 3 U phosphoglucomutase (Sigma-Aldrich) and 0.26 U/mg IAGlc synthase. The change of the NADPH/H^+^ absorbance was monitored at 340 nm using a Shimadzu UV-160A spectrophotometer (Shimadzu, Japan). The enzyme activity was calculated using the absorption coefficient 6220 M^−1^ cm^−1^ for NADH/H^+^.

### 3.3. Kinetic Studies

#### 3.3.1. The Effect of pH on IAGlc Synthase Activity

The effect of pH on the catalytic activity of the recombinant IAGlc synthase was investigated in 25 mM HEPES buffer ranging from pH 5.8 to 8.8 (pH 5.8; 6.2; 6.6; 6.8; 7.0; 7.4; 7.6; 8.0; 8.2; 8.6; 8.8) using the radioactivity method in standard conditions. The reaction was stopped after 30 min incubation at 30 °C by drying 4 μL of aliquots on a Silica Gel F_260_ TLC plate (Merck). The rest of the assay was performed as described above.

#### 3.3.2. The Effect of Divalent Cations on IAGlc Synthase Activity

The effect of divalent ions on the catalytic activity of recombinant IAGlc synthase was investigated in a total volume of 8 μL containing 25 mM HEPES buffer, pH 7.4, 7.5 mM UDPG, 4 mM IAA and 0.016 μCi [2′-^14^C]IAA (55 mCi mmol^−1^, Hartmann Analytic GmBH, Braunschweig, Germany) with 3 μL of the enzyme preparation. The reaction mixture contained 5 mM MgCl_2_, 5 mM CaCl_2_, 5 mM MnCl_2_, 5 mM EDTA or no ions (control). The reaction was stopped after 15 min incubation at 30 °C by drying 4 μL of aliquots on a Silica Gel F_260_ TLC plate (Merck). The rest of the assay was performed as described above.

#### 3.3.3. The Effect of Modulators on IAGlc Synthase Activity

The effect of modulators on the catalytic activity of recombinant IAGlc synthase was investigated in a total volume of 8 μL containing 25 mM HEPES buffer, pH 7.4, 7.5 mM UDPG, 4 mM IAA, 0.016 μCi [2′-^14^C]IAA (55 mCi mmol^−1^, Hartmann Analytic GmBH, Germany) and 2.5 mM MgCl_2_ with 3 μL of the enzyme preparation. The reaction mixture contained one of the potential modulators: 1 mM ATP; 10 mM oxidized glutathione (GSSG); 10 mM reduced glutathione (GSH); 4 mM glucose-1-phosphate (Glc-1-P); 4 mM pyrophosphate (PPi); 0.1 mM IAA–Asp or 1 mM IAA–Asp. The control did not contain any modulators. The reaction was stopped after 15 min incubation at 30 °C by drying 4 μL of aliquots on a Silica Gel F_260_ TLC plate (Merck). The rest of the assay was performed as described above.

#### 3.3.4. The Effect of Phytohormones on IAGlc Synthase Activity

The effect of phytohormones on the catalytic activity of recombinant IAGlc synthase was investigated in a total volume of 8 μL containing 25 mM HEPES buffer, pH 7.4, 7.5 mM UDPG, 4 mM IAA, 0.016 μCi [2′-^14^C]IAA (55 mCi mmol^−1^, Hartmann Analytic GmBH, Germany) and 2.5 mM MgCl_2_ with 3 μL of the enzyme preparation. The reaction mixture contained one of the following phytohormones: 1 mM phenylacetic acid (PAA); 1 mM 2,4-dichlorophenoxyacetic acid (2,4-D); 1 mM Picloram; 1 mM Dicamba; 1 mM gibberellic acid (GA_3_) or 1 mM kinetin. The control did not contain any additional phytohormones. The reaction was stopped after 15 min incubation at 30 °C by drying 4 μL of aliquots on a Silica Gel F_260_ TLC plate (Merck). The rest of the assay was performed as described above.

#### 3.3.5. Determination of Kinetic Parameters for UDPG

The effect of UDPG concentration on the IAGlc synthase activity was analyzed in a total volume of 8 μL containing 25 mM HEPES buffer, pH 7.4, 3 mM IAA and 0.016 μCi [2′-^14^C]IAA (55 mCi mmol^−1^, Hartmann Analytic GmBH, Germany) with 3 μL of the enzyme preparation with different UDPG concentrations (0.5–20 mM). The reaction was stopped after 15 min incubation at 30 °C by drying 4 μL of aliquots on a Silica Gel F_260_ TLC plate (Merck). The rest of the assay was performed as described above. The *V_max_* and *K_M_* parameters were calculated using a Hanes–Woolf plot ([S]/*v* = f_([S])_) and verified by the Michaelis–Menten equation using SigmaPlot 11.0 (Systat Software Inc, San Jose, California, USA).

#### 3.3.6. Determination of Kinetic Parameters for IAA

The effect of IAA concentration on the IAGlc synthase (4) activity was analyzed using the spectrophotometric method [16] as the release of UDP using a coupled enzyme assay containing pyruvate kinase (5) and lactate dehydrogenase (6) according to the following equations:

IAA + UDPG ↔ IAGlc + UDP(4)

UDP + phosphoenolpyruvate ↔ UTP + pyruvate(5)

Pyruvate + NADH/H^+^ → l-lactate + NAD^+^ (6)

The reaction mixture in the total volume of 1.0 mL contained 50 mM HEPES–NaOH, pH 7.4, 2.5 mM MgCl_2_, 2 mM PEP, 4 mM UDPG, IAA (0.5–4 mM), 0.13 mM NADH/H^+^, 3 U pyruvate kinase from rabbit muscles (Sigma-Aldrich), 4 U l-lactate dehydrogenase from rabbit muscles (Sigma-Aldrich) and 0.26 U/mg IAGlc synthase. The change of the NADH/H^+^ absorbance was monitored at 340 nm using a Shimadzu UV-160A spectrophotometer (Shimadzu, Japan). The enzyme activity was calculated using the absorption coefficient 6220 M^−1^ cm^−1^ for NADH/H^+^. The *V_max_* and *K_M_* parameters were calculated using a Hanes–Woolf plot and verified by the Michaelis–Menten equation using SigmaPlot 11.0 (Systat Software). The effect of IAA–Asp on the *K_M_* and *V_max_* values was analyzed as described above using the reaction mixtures containing 0.5–4 mM IAA and additionally 0.1 mM IAA–Asp.

#### 3.3.7. Determination of Substrate Specificity of IAGlc Synthase

The substrate specificity of the IAGlc synthase (7) was analyzed using the spectrophotometric method [16] as the release of UDP using a coupled enzyme assay containing pyruvate kinase (8) and lactate dehydrogenase (9) according to the following equations:

substrate + UDPG ↔ substrate–Glc + UDP(7)

UDP + phosphoenolpyruvate ↔ UTP + pyruvate(8)

Pyruvate + NADH/H^+^ → l-lactate + NAD^+^ (9)

The reaction mixture in the total volume of 1.0 mL contained 50 mM HEPES–NaOH, pH 7.4, 2.5 mM MgCl_2_, 2 mM PEP, 4 mM UDPG, 0.13 mM NADH/H^+^, 3 U pyruvate kinase from rabbit muscles (Sigma-Aldrich), 4 U l-lactate dehydrogenase from rabbit muscles (Sigma-Aldrich), 4.3 µg ZmIAGlc synthase and 3 mM sugar acceptor (IAA, IBA, IPA, IPyA, PAA, 2,4-D, Dicamba, Picloram or tryptophan). The change of the NADH/H^+^ absorbance was monitored at 340 nm using a Shimadzu UV-160A spectrophotometer (Shimadzu, Japan). The enzyme activity was calculated using the absorption coefficient 6220 M^−1^ cm^−1^ for NADH/H^+^.

#### 3.3.8. Inhibition of IAGlc by ATP and Glucose-1-Phosphate

The effect of ATP on the IAGlc synthase activity was studied in the standard reaction mixture with ATP at the final concentration of 0–1 mM. The effect of glucose-1-phosphate (Glc-1-P) on the IAGlc synthase activity was studied in the standard reaction mixture with Glc-1-P at the final concentration of 0–4 mM. The reaction was stopped after 15 min incubation at 30 °C by drying 4 μL of aliquots on a Silica Gel F_260_ TLC plate (Merck). The rest of the assay was performed as described above.

#### 3.3.9. Inhibition of IAGlc Synthase by 2,4-D

The effect of 2,4-D on the IAGlc synthase activity was determined using the radioactivity assay in the standard reaction mixture with IAA concentrations of 1.95 mM and 3.0 mM in the presence of 0–625 μM 2,4-D. The inhibition kinetics were determined using a Dixon plot (1/*v* = f_([I])_). The inhibition constant (*K_i_*) value was calculated using a Dixon plot. The IC_50_ value was calculated through graphical analysis. The resulting data were fit into the following equation: *K_M_^app^ = K_m_ (1 + [I]/K_i_).* Calculations were performed using SigmaPlot 11.0 (Systat Software).

### 3.4. Western Blot Analysis

For Western blot analysis, the proteins were separated by SDS-PAGE according to the method of Ogita and Markert [42] and transferred onto a Protran BA 83 nitrocellulose membrane (Whatman, GmBH, Maidstone, UK) by the wet system (Bio-Rad) (200 mA, 90 V for 1 h at 4 °C) in 10 mM CAPS/NaOH buffer, pH 11.0, containing 10% (*v*/*v*) ethanol. The Spectra^TM^ multicolor broad range protein ladder (10–260 kDa) (Thermo Scientific, Waltham, Massachusetts, USA) was used as a molecular mass standard. After blocking in TBS (Tris Buffered Saline) buffer containing 5% (*w*/*v*) nonfat dry milk, the membrane was incubated with monoclonal anti-polyHistidine antibodies produced in mice (1:12,000) (Sigma-Aldrich). The proteins were detected with goat anti-mouse IgG antibodies conjugated to alkaline phosphatase (1:1000) (Sigma-Aldrich) and visualized using NBT/BCIP (nitro blue tetrazolium/5-bromo-4-chloro-3-indolyl-phosphate) tablets (Roche, Bazylea, Sweden).

### 3.5. Protein Determination

Protein concentration was determined with the Bradford method [45] using bovine serum albumin as a standard.

### 3.6. In Silico Sequence Analysis

Amino acid sequences of maize IAGlc synthase (ZmIAGLU, NCBI Reference Sequence Database, accession number NP_001105326.1), rice OsIAGT1 (GenBank, accession number BAS85861.1) and *A. thaliana* glucosyltransferases UGT74D1 (UniProtKB/Swiss-Prot, accession number Q9SKC5.1), UGT74E2 (GenBank, accession number ABJ17124.1), UGT75D1 (UniProtKB/Swiss-Prot, accession number O23406.2), UGT76F1 (UniProtKB/Swiss-Prot, accession number Q9M051.1) and UGT84B1 (GenBank, accession number AAO63432.1) were aligned using Clustal Omega (https://www.ebi.ac.uk/Tools/msa/clustalo/, access date: 1 February 2021). Identity of these sequences was calculated also using Clustal Omega. Prediction of disulfide bonds was performed using the DiANNA 1.1 web Server (http://clavius.bc.edu/~clotelab/DiANNA/, accessed on 2 February 2021).

### 3.7. Homology Modeling and Molecular Docking

The secondary and three-dimensional structure of IAGlc synthase was predicted using the iterative threading assembly refinement (I-TASSER) server [36,37,38,39]. The five models were generated by the I-TASSER server and the best model was selected on the basis of sequence identity and the estimated global accuracy of the model (C-Score = 0.77, TM-score = 0.82 ± 0.09, and RMSD = 5.5 ± 3.5 Å). The obtained 3D structure of IAGlc synthase was used in the molecular docking process.

The three-dimensional structure of UDPG, ATP and Glc-1-P were downloaded from the Protein Data Bank (http://www.rcsb.org/pdb/home/home.do, accessed on 20 February 2021). Prediction of molecular interactions of IAGlc synthase with ligands was made using the SwissDock web service with default server values [40]. The results were visualized in UCFS Chimera [46]. The binding interactions between the substrate (UDPG) or modulators (ATP, Glc-1-P) and IAGlc synthase were simulated using the docking method implemented in AutoDock 4.2 along with Autodock Tools [41]. A semiflexible docking protocol was used. The protein molecule was kept rigid, whereas ligands were kept as flexible molecules. The grid box (72 × 70 × 62) with 0.375 Å was fixed around the active site of the protein (PSPG motif, TRP314, CYS342, PRO343, HIS359, TRP362, ASN363, SER364, GLU367, ASP383 and GLN384) [4]. The center of the grid box was set at points 70.199, 65.543, 63.479. Then, the grid maps were precalculated using Autogrid, one map for each atom type present in the docked ligand. The Lamarckian genetic algorithm (LGA) was used for searching the best conformation of complexes. The docking parameters were set to the default value. The position with the best binding affinity was visualized using Pymol [47].

### 3.8. Statistical Analysis

All data are presented as the means ± SD for three biological repetitions of each experiment (*n* = 3).

## 4. Conclusions

Our research provides a biochemical characterization of IAGlc synthase—the enzyme modulating the IAA biological activity by its conjugation to glucose. The enzyme is highly specific towards IAA; however, it also utilizes other natural auxins (IBA, IPA and IPyA) as glucose acceptors, though with less efficiency. IAGlc synthase activity is modulated by a number of compounds. 2,4-D, a synthetic auxinic herbicide, acts as a competitive inhibitor of the synthase. We also report that IAA–Asp, another conjugated form of IAA, acts as an activator of IAGlc synthase. Such a role can suggest that auxin conjugate synthesis is a complex process regulated not only on the expression level, but also on the protein level by products of different conjugation pathways. We also report unique modulatory properties of ATP and glucose-1-phosphate whose action is concentration-dependent. At lower concentrations, both compounds act as activators of IAGlc synthase, however, a high concentration of these compounds strongly inhibits this enzyme. Molecular docking revealed that both ATP and glucose-1-phosphate, similar to the glucose donor—UDPG, can bind to the PSPG motif of IAGlc synthase. However, more potential binding sites for these two compounds are present in the enzyme structure. We suggest that IAGlc synthase may contain more than one binding site for these two modulatory compounds, one site being responsible for the activating effect of the modulator and another, possibly the PSPG motif, causing abolishment of synthase activity.

## Figures and Tables

**Table 1:** Substrate specificity of recombinant IAGlc synthase. Enzyme activity was determined using the spectrophotometric method. Relative activity (%) was calculated taking the IAGlc synthase activity for IAA (nmol min^−1^ mg^−1^ protein) as 100%. IAA—indole-3-acetic acid; IBA—indole-3-butyric acid; IPA—indole-3-propionic acid; IPyA—indole-3-pyruvic acid; PAA—phenylacetic acid; 2,4-D—2,4-dichlorophenoxyacetic acid.

Substrate	IAGlc Synthase (nmol min^−1^ mg^−1^ protein)	Relative Activity (%)
IAA	210.46 ± 0.5	100
IBA	129.53 ± 1.04	66
IPA	104.65 ± 3.8	49.7
IPyA	5.81 ± 0.2	2.76
PAA	0	0
2,4-D	0	0
Dicamba	0	0
Picloram	0	0
Tryptophan	0	0

**Table 2:** Kinetic parameters of recombinant maize IAGlc synthase.

Substrate	Cosubstrate	*K_M_* (mM)	*V_max_* (nmol min^−1^ mg^−1^ protein)
IAA	4 mM UDPG	0.8 ± 0.1	519 ± 27
UDPG	3 mM IAA	0.7 ± 0.09	243 ± 18

The *K_M_* and *V_max_* parameters were calculated using a Hanes–Woolf plot and confirmed by the Michaelis–Menten equation. All values are expressed as the means ± SD (*n* = 3).

**Table 3:** Inhibition of IAGlc synthase by 2,4-dichlorophenoxyacetic acid (2,4-D).

Inhibitor	Inhibition Type	*K_i_* (μM)	IC_50_ (μM)	*K_M_^app^* (mM)
2,4-D	Competitive (IAA)	117 ± 10	350 ± 28 (for 3 mM IAA) 439 ± 33 (for 1.95 mM IAA)	1.22 ± 0.08 (1.53-fold decrease of affinity)

**Table 4:** Sequence identities of IAA glucosyltransferases. The sequence identities were calculated using Clustal Omega.

UGT	Sequence Identity with Maize IAGlc Synthase	PSPG Motif Identity with Maize IAGlc Synthase
UGT76F1	27.55%	59.09%
UGT84B1	34.10%	61.36%
UGT75D1	35.27%	65.91%
UGT74D1	39.82%	65.91%
UGT74E2	40.41%	75.00%
OsIAGT1	69.35%	88.64%

## Data Availability

The data presented in this study are available in article.

## Abbreviations

1-*O*-IAGlc	1-*O*-indole-3-acetyl-β-d-glucose
2,4-D	2,4-dichlorophenoxyacetic acid
ATP	adenosine triphosphate
GA	gibberellic acid
GSH	reduced glutathione
GSSG	oxidized glutathione
GT	glycosyltransferase
IAA	indole-3-acetic acid
IBA	indole-3-butyric acid
IPA	indole-3-propionic acid
IPyA	indole-3-pyruvic acid
IPTG	isopropyl-1-thio-β-d-galactopyranoside
NAA	naphthaleneacetic acid
OxIAA	oxindole-3-acetic acid
PAA	phenylacetic acid
PEP	phosphoenolopyruvate
PP_i_	pyrophosphate
PSPG	plant secondary product glycosyltransferase
UDP	uridine diphosphate
UDPG	uridine diphosphate glucose
UGT	uridine diphosphate glycosyltransferase
*Zm-IAGlc*	UDPG-dependent IAA glucosyltransferase gene

## References

[1] Breton C., Fournel-Gigleux S., Palcic M.M. (2012). Recent structures, evolution and mechanisms of glycosyltransferases. Curr. Opin. Struct. Biol..

[2] Ross J., Li Y., Lim E.K., Bowles D.J. (2001). Higher plant glycosyltransferases. Genome Biol..

[3] MacKenzie P.I., Owens I.S., Burchell B., Bock K.W., Bairoch A., Belanger A., Gigleux S.F., Green M., Hum D.W., Iyanagi T. (1997). The UDP glycosyltransferase gene superfamily: Recommended nomenclature update based on evolutionary divergence. Pharmacogenetics.

[4] Osmani S.A., Bak S., Møller B.L. (2009). Substrate specificity of plant UDP-dependent glycosyltransferases predicted from crystal structures and homology modeling. Phytochemistry.

[5] Yonekura-Sakakibara K., Hanada K. (2011). An evolutionary view of functional diversity in family 1 glycosyltransferases. Plant J..

[6] Korasick D.A., Enders T.A., Strader L.C. (2013). Auxin biosynthesis and storage forms. J. Exp. Bot..

[7] Bajguz A., Piotrowska A. (2009). Conjugates of auxin and cytokinin. Phytochemistry.

[8] Ludwig-Müller J. (2011). Auxin conjugates: Their role for plant development and in the evolution of land plants. J. Exp. Bot..

[9] Michalczuk L., Bandurski R.S. (1980). UDP-glucose: Indoleacetic acid glucosyl transferase and indoleacetyl-glucose: Myo-inositol indoleacetyl transferase. Biochem. Biophys. Res. Commun..

[10] Kęsy J.M., Bandurski R.S. (1990). Partial purification and characterization of indol-3-ylacetylglucose: Myo-inositol in-dol-3-ylacetyltransferase (indoleacetic acid-inositol synthase). Plant. Physiol..

[11] Kowalczyk S., Jakubowska A., Zielińska E., Bandurski R.S. (2003). Bifunctional indole-3-acetyl transferase catalyses synthesis and hydrolysis of indole-3-acetyl-myo-inositol in immature endosperm of *Zea mays*. Physiol. Plant..

[12] Leźnicki A.J., Bandurski R.S. (1988). Enzymic synthesis of indole-3-acetyl-1-O-β-D-glucose. I. Partial purification and characterization of the enzyme from *Zea mays*. Plant. Physiol..

[13] Leznicki A.J., Bandurski R.S. (1988). Enzymic Synthesis of Indole-3-Acetyl-1-O-β-d-Glucose. II. Metabolic Characteristics of the Enzyme. Plant Physiol..

[14] Kowalczyk S., Bandurski R.S. (1991). Enzymic synthesis of 1-O-(indol-3-ylacetyl)-β-d-glucose. Purification of the enzyme from *Zea mays*, and preparation of antibodies to the enzyme. Biochem. J..

[15] Szerszen J.B., Szczyglowski K., Bandurski R.S. (1994). iaglu, a gene from *Zea mays* involved in conjugation of growth hormone indole-3-acetic acid. Science.

[16] Jackson R.G., Lim E.K., Li Y., Kowalczyk M., Sandberg G., Hoggett J., Ashford D.A., Bowles D.J. (2001). Identification and Biochemical Characterization of an Arabidopsis Indole-3-acetic Acid Glucosyltransferase. J. Biol. Chem..

[17] Tognetti V.B., Van Aken O., Morreel K., Vandenbroucke K., Van De Cotte B., De Clercq I., Chiwocha S., Fenske R., Prinsen E., Boerjan W. (2010). Perturbation of Indole-3-Butyric Acid Homeostasis by the UDP-Glucosyltransferase UGT74E2 Modulates Arabidopsis Architecture and Water Stress Tolerance. Plant Cell.

[18] Tanaka K., Hayashi K.I., Natsume M., Kamiya Y., Sakakibara H., Kawaide H., Kasahara H. (2014). UGT74D1 Catalyzes the Glucosylation of 2-Oxindole-3-Acetic Acid in the Auxin Metabolic Pathway in Arabidopsis. Plant Cell Physiol..

[19] Zhang G.Z., Jin S.H., Jiang X.Y., Dong R.R., Li P., Li Y.J., Hou B.K. (2015). Ectopic expression of UGT75D1, a glycosyltransferase preferring indole-3-butyric acid, modulates cotyledon development and stress tolerance in seed germination of *Arabidopsis thaliana*. Plant Mol. Biol..

[20] Chen L., Huang X.X., Zhao S.M., Xiao D.W., Xiao L.T., Tong J.H., Wang W.S., Li Y.J., Ding Z., Hou B.K. (2020). IPyA glucosylation mediates light and temperature signaling to regulate auxin-dependent hypocotyl elongation in Arabidopsis. Proc. Natl. Acad. Sci. USA.

[21] Ostrowski M., Hetmann A., Jakubowska A. (2015). Indole-3-acetic acid UDP-glucosyltransferase from immature seeds of pea is involved in modification of glycoproteins. Phytochemistry.

[22] Liu Q., Chen T.T., Xiao D.W., Zhao S.M., Lin J.S., Wang T., Li Y.J., Hou B.K. (2019). OsIAGT1 is a glucosyltransferase gene involved in the glucose conjugation of auxin in rice. Rice.

[23] Ostrowski M., Ciarkowska A., Dalka A., Wilmowicz E., Jakubowska A. (2020). Biosynthesis pathway of indole-3-acetyl-myo-inositol during development of maize (*Zea mays* L.) seeds. J. Plant Physiol..

[24] Choi M.S., Koh E.B., Woo M.O., Piao R., Oh C.S., Koh H.J. (2012). Tiller formation in rice is altered by overexpression of *OsIAGLU* gene encoding an IAA-conjugating enzyme or exogenous treatment of free IAA. J. Plant Biol..

[25] Yu X.L., Wang H.Y., Leung D.W.M., He Z.D., Zhang J.J., Peng X.X., Liu E.E. (2019). Overexpression of *OsIAAGLU* reveals a role for IAA–glucose conjugation in modulating rice plant architecture. Plant Cell Rep..

[26] Jin S., Hou B., Zhang G. (2021). The ectopic expression of Arabidopsis glucosyltransferase UGT74D1 affects leaf positioning through modulating indole-3-acetic acid homeostasis. Sci. Rep..

[27] Michalczuk L., Bandurski R.S., Corcuera L.J., Kowalczyk S., Keglevic D., Pokorný M., Gazarian I.G., Lagrimini L.M., Mellon F.A., Naldrett M.J. (1982). Enzymic synthesis of 1-O-indol-3-ylacetyl-β-d-glucose and indol-3-ylacetyl-myo-inositol. Biochem. J..

[28] Gasser B., Saloheimo M., Rinas U., Dragosits M., Rodríguez-Carmona E., Baumann K., Giuliani M., Parrilli E., Branduardi P., Lang C. (2008). Protein folding and conformational stress in microbial cells producing recombinant proteins: A host comparative overview. Microb. Cell Factories.

[29] Jin S.H., Ma X.M., Han P., Wang B., Sun Y.G., Zhang G.Z., Li Y.J., Hou B.K. (2013). UGT74D1 Is a Novel Auxin Glycosyltransferase from *Arabidopsis thaliana*. PLoS ONE.

[30] Ostrowski M., Świdziński M., Ciarkowska A., Jakubowska A. (2014). IAA-amido synthetase activity and *GH3* expression during development of pea seedlings. Acta Physiol. Plant..

[31] Kowalczyk S., Jakubowska A., Bandurski R.S. (2002). 1-Naphtalene acetic acid induces indole-3-ylacetylglucose synthase in *Zea mays* seedling tissue. Plant Growth Regul..

[32] Aoi Y., Hira H., Hayakawa Y., Liu H., Fukui K., Dai X., Tanaka K., Hayashi K.-I., Zhao Y., Kasahara H. (2020). UDP-glucosyltransferase UGT84B1 regulates the levels of indole-3-acetic acid and phenylacetic acid in Arabidopsis. Biochem. Biophys. Res. Commun..

[33] Berlec A., Štrukelj B. (2013). Current state and recent advances in biopharmaceutical production in *Escherichia coli*, yeasts and mammalian cells. J. Ind. Microbiol. Biotechnol..

[34] Caputi L., Malnoy M., Goremykin V., Nikiforova S., Martens S. (2012). A genome-wide phylogenetic reconstruction of family 1 UDP-glycosyltransferases revealed the expansion of the family during the adaptation of plants to life on land. Plant J..

[35] Ono E., Homma Y., Horikawa M., Kunikane-Doi S., Imai H., Takahashi S., Kawai Y., Ishiguro M., Fukui Y., Nakayama T. (2010). Functional Differentiation of the Glycosyltransferases That Contribute to the Chemical Diversity of Bioactive Flavonol Glycosides in Grapevines (*Vitis vinifera*). Plant Cell.

[36] Zhang Y. (2008). I-TASSER server for protein 3D structure prediction. BMC Bioinform..

[37] Roy A., Kucukural A., Zhang Y. (2010). I-TASSER: A unified platform for automated protein structure and function prediction. Nat. Protoc..

[38] Yang J., Roy A., Zhang Y. (2013). Protein–ligand binding site recognition using complementary binding-specific substructure comparison and sequence profile alignment. Bioinformatics.

[39] Yang J., Yan R., Roy A., Xu D., Poisson J., Zhang Y. (2015). The I-TASSER Suite: Protein structure and function prediction. Nat. Methods.

[40] Grosdidier A., Zoete V., Michielin O. (2011). SwissDock, a protein-small molecule docking web service based on EADock DSS. Nucleic Acids Res..

[41] Morris G.M., Huey R., Lindstrom W., Sanner M.F., Belew R.K., Goodsell D.S., Olson A.J. (2009). AutoDock4 and AutoDockTools4: Automated docking with selective receptor flexibility. J. Comput. Chem..

[42] Ogita Z.I., Markert C.L. (1979). A miniaturized system for electrophoresis on polyacrylamide gels. Anal. Biochem..

[43] Shevchenko A., Wilm M., Vorm O., Mann M. (1996). Mass Spectrometric Sequencing of Proteins from Silver-Stained Polyacrylamide Gels. Anal. Chem..

[44] Ehmann A. (1977). The Van-Urk-Salkowski reagent: A sensitive and specific chromogenic reagent for silica gel thin-layer chromato-graphic detection and identification of indole derivatives. J. Chromatogr..

[45] Bradford M.M. (1976). A rapid and sensitive method for the quantitation of microgram quantities of protein utilizing the principle of protein-dye binding. Anal. Biochem..

[46] Pettersen E.F., Goddard T.D., Huang C.C., Couch G.S., Greenblatt D.M., Meng E.C., Ferrin T.E. (2004). UCSF Chimera: A visualization system for exploratory research and analysis. J. Comput. Chem..

[47] DeLano W.L. (2002). Pymol: An open-source molecular graphics tool. CCP4 Newsl. Protein Crystallogr..

